# Doxorubicin-Loaded Fungal-Carboxymethyl Chitosan Functionalized Polydopamine Nanoparticles for Photothermal Cancer Therapy

**DOI:** 10.3390/pharmaceutics15041281

**Published:** 2023-04-19

**Authors:** Maduru Suneetha, Hyeonjin Kim, Sung Soo Han

**Affiliations:** 1 School of Chemical Engineering, Yeungnam University, Gyeongsan 38541, Republic of Korea; msunithachem@gmail.com (M.S.); hyeonjin3438@naver.com (H.K.); 2 Research Institute of Cell Culture, Yeungnam University, Gyeongsan 38541, Republic of Korea

**Keywords:** polydopamine, fungal-carboxymethyl chitosan, photothermal property, drug release, cancer therapy

## Abstract

In this work, we synthesized doxorubicin-loaded fungal-carboxymethyl chitosan (FC) functionalized polydopamine (Dox@FCPDA) nanoparticles for improved anticancer activity via photothermal drug release. The photothermal properties revealed that the FCPDA nanoparticles with a concentration of 400 µg/mL produced a temperature of about 61.1 °C at 2 W/cm^2^ laser illumination, which is more beneficial for cancer cells. Due to the hydrophilic FC biopolymer, the Dox was successfully encapsulated into FCPDA nanoparticles via electrostatic interactions and pi-pi stacking. The maximum drug loading and encapsulation efficiency were calculated to be 19.3% and 80.2%, respectively. The Dox@FCPDA nanoparticles exhibited improved anticancer activity on HePG2 cancer cells when exposed to an NIR laser (800 nm, 2 W/cm^2^). Furthermore, the Dox@FCPDA nanoparticles also improved cellular uptake with HepG2 cells. Therefore, functionalizing FC biopolymer with PDA nanoparticles is more beneficial for drug and photothermal dual therapeutic properties for cancer therapy.

## 1. Introduction

Light stimulation therapy, which includes photothermal therapy (PTT) and photodynamic therapy, is currently one of the most promising cancer treatments. In PTT, when irradiated with light, nanoparticles can produce heat that can kill cancer cells [1]. The combination of chemo and photothermal therapy is more advantageous when photothermal nanoparticles are combined with chemotherapeutic agents that can destroy cancerous cells via synergistic action (chemo and photothermal property) [2,3]. Recently, a wide range of near-infrared (NIR) active nanoparticles have been utilized for photothermal therapy, such as metal-based or metal sulfide and other carbon-based materials [2,3,4]. Among these, the combination of photothermal agents decorated with polymers has been attractive for loading chemotherapeutic agents and is more beneficial for combined chemo and photothermal therapy for cancer [5].

Among various kinds of materials, polydopamine (PDA) is attractive in photothermal and chemotherapy because of its structure, which is similar to melanin and has excellent biocompatibility [6]. PDA can easily produce heat when irradiated with NIR light. It can be used as an excellent photothermal therapeutic property. Although PDA-based nanoformulations have been extensively developed for PTT for cancer therapy, the surviving cancer cells could lead to metastases and a return of the disease due to the partial ablation of tumor tissues. Due to the limited permeability of combined photothermal nanoagents in tumor tissues, therapeutic agents are distributed unevenly throughout cancer tissues [6]. To address this issue, novel photothermal therapeutic nanoparticle design is required. Therefore, the polymer-modified PDA nanoparticles improve drugs’ retention ability and the prolonged release of drugs [7,8]. These PDA NPs, however, displayed considerable toxicity to healthy cells and tissues due to their non-specificity. Two efficient methods for resolving the aforementioned issue include creating responsive PDA-based nanoformulations and grafting some targeting units on the PDA-based nanoformulations [8]. With regard to PTT with PDA, the recent literature has focused on functionalizing PDA-based nanoparticles to enhance their drug delivery efficiency, targeting ability, and therapeutic efficacy. One approach involves using ligands or antibodies to functionalize the surface of polydopamine-based nanoparticles to improve their targeting ability. For example, targeting ligands such as folic acid and aptamers have been conjugated to the surface of PDA-based nanoparticles to selectively target cancer cells that overexpress specific receptors [9,10,11,12]. Another approach involves incorporating additional functional groups or moieties onto the surface of PDA-based nanoparticles to enhance their drug loading capacity or promote controlled drug release [13,14,15]. For example, thiol or amine groups can be incorporated onto the surface of PDA-based nanoparticles to enhance their drug loading capacity or enable conjugation of other therapeutic agents or targeting moieties [16,17,18]. In addition to surface functionalization, recent studies have explored the use of PDA-based nanoparticles in combination with other therapeutic modalities such as photothermal therapy and gene therapy [13,14,15]. For example, PDA-based nanoparticles have been used as carriers for both chemotherapy drugs and photothermal agents to enable synergistic chemo-photothermal therapy. Overall, functionalized PDA-based nanoparticles show great promise for improving cancer drug delivery and overcoming some of the limitations associated with conventional drug delivery approaches. However, further research is needed to optimize their design and evaluate their long-term safety and efficacy in clinical settings.

Natural polymers such as chitosan, sodium alginate, starch, and pectin have been utilized for DDSs to improve the bioavailability of many potential drugs [19]. Among these, chitosan (CS) is one of the most promising for DDSs [20]. It has mucoadhesive, permeation-enhancing, in situ gelling, and efflux pump inhibitory effects because of its cationic nature. Additionally, a controlled medication release can be accomplished via ionic interactions, and nanoparticulate delivery systems for siRNA and pharmaceuticals based on DNA can be created [21]. Carboxymethyl chitosan (CMCS) is a modified CS that also attracted drug delivery in cancer therapy. It has carboxylic and cationic amino groups, which are responsible for the loading of drugs via the formation of H-bonding and complexation and which can deliver the drug molecules easily in the tumor microenvironment [22]. As of late, it has been shown that CMCS derived from non-animal sources of fungal mushrooms (FC) has outstanding physiological and biological characteristics, including increased water solubility in a wide range of pH solutions, biodegradability, and excellent biocompatibility. Therefore, FC has been utilized for the preparation of nanocomposites, films, and hydrogels for biomedical applications [23,24,25]. 

By considering the excellent properties of FC and the PTT ability of PDA, herein we developed FCPDA nanoparticles for loading Dox as a chemotherapeutic agent. The presence of amino functional groups on FC biopolymer can easily be covalently bonded to the DOPA structure during the formation of PDA, thereby stabilizing the FCPDA nanoparticles. By considering the excellent properties of FC, the FCPDA nanoparticles are easily allowed to encapsulate with Dox drug via the formation of multiple bonds such as H-bond, complexation, and pi-pi stacking. The resulting Dox@FCPDA nanoparticle was studied for its combined chemo and photothermal properties for cancer therapy.

## 2. Materials and Methods

### 2.1. Materials

The Endovision Company (Daegu, Republic of Korea) kindly provided FC (originated from Agaricus Bisporous Mushroom) with MW = 200–2000 KDa (viscosity 20–1000 cps with deacetylation 80–98%). DA was purchased from Sigma Aldrich Company, Seoul, Republic of Korea. Ammonia (25–28%) solution was purchased from Dae-Jung chemical metal Co., Ltd., Siheung-si, Gyeonggi-Do, Republic of Korea.

### 2.2. Synthesis of FCPDA Nanoparticles

First, 350 µL of aqueous ammonia solution (25–28%) was combined with 3.2 mL of ethanol and 7.2 mL of double distilled water (DDW). Then, the solution of FC (20 mg) and DA (40 mg) in one milliliter of DDW was prepared. Finally, the FC and DA solution mixture was slowly added to the aforementioned solution. The solution was stirred at 600 rpm for 20 min at open-air conditions, and the reaction continued for 24 h. Finally, the solution was centrifuged at 4000 rpm for 10 min, subsequently washed with DDW, and centrifugation was repeated 3 times at room temperature. Finally, the FCPDA nanoparticles were freeze-dried for 24 h.

### 2.3. Preparation of Dox@FCPDA Nanoparticles and Encapsulation Efficiency

Dox@FCPDA nanoparticles were prepared by immersing FCPDA nanoparticles in Dox solutions. A total of 10 mg of nanoparticles (FCPDA nanoparticles) were dispersed in 2 mg of Dox in 5 mL of DDW. The dispersion was stirred for 24 h and then centrifuged at 10,000 rpm for 10 min at room temperature under dark conditions. The recovered nanoparticles were washed with DDW and further centrifuged at 10,000 rpm for 10 min at room temperature. The supernatant solution was analyzed for UV. The drug loading and encapsulation was calculated as follows.

$$\mathrm{DL}\;\left(\%\right)=\frac{\mathrm{Weight}\;\mathrm{of}\;\mathrm{Dox}\;\mathrm{in}\;\mathrm{the}\;\mathrm{FC}-\mathrm{PDA}\;\mathrm{nanoaprticles}}{\mathrm{Weight}\;\mathrm{of}\;\mathrm{FC}-\mathrm{PDA}\;\mathrm{recovered}\;\mathrm{nanoaprticles}}\times 100$$

$$\mathrm{EE}\;\left(\%\right)=\frac{\mathrm{Weight}\;\mathrm{of}\;\mathrm{Dox}\;\mathrm{in}\;\mathrm{the}\;\mathrm{FC}-\mathrm{PDA}\;\mathrm{nanoaprticles}}{\mathrm{Weight}\;\mathrm{of}\;\mathrm{FC}-\mathrm{PDA}\;\mathrm{recovered}\;\mathrm{nanoaprticles}}\times 100$$

The Dox release from FCPDA nanoparticles was analyzed in PBS solutions (pH 7.4 and 5.0) at 37 °C. A total of 1 mg of Dox@FCPDA nanoparticles were dispersed in a vial with 2 mL of releasing media and incubated in an orbital shaker with 100 rpm at 37 °C. At a predetermined time, the vial was centrifuged for 2 min at 5000 rpm. A similar volume of fresh medium was added once the supernatant solution was withdrawn. Using UV-Vis absorption spectrophotometry, the released Dox was examined at 487 nm. Additionally, the Dox release from FCPDA nanoparticles was carried out for 10 min of exposure to NIR laser (800 nm) operating at 2 W/cm^2^.

### 2.4. Characterization

FTIR spectra of FCPDA, Dox, and Dox@FCPDA nanoparticles were analyzed by using a Perkin Elmer instrument (FTIR-410, Shelton, WA, USA) under transmittance mode between 500 and 4000 cm^−1^. Using field-emission transmission electron microscopy, the morphology of the FCPDA nanoparticles was examined (FE-TEM, Tecnai G2 F20, Hillsboro, OR, USA). The hydrodynamic diameter of the products was measured using dynamic light scattering (DLS, Malvern Zetasizer Nano-ZS, Worcestershire, UK). The UV-Vis-NIR absorption spectra of FCPDA nanoparticles with various concentrations were analyzed using a Shimadzu-2600 instrument (Kyoto, Japan) and scanned between 250 and 1000 nm. Using a thermal camera (Peak-Tech-3450, Bangkok, Thailand), infrared thermal images were captured and evaluated. The drug release profile of the Dox@FCPDA nanoparticles was determined via UV-Vis absorption spectrophotometry (Shimadzu-2600, (Kyoto, Japan)) scanned between 350 and 600 nm.

### 2.5. Photothermal Properties

The photothermal effect of the FCPDA nanoparticles under NIR laser illumination was performed using a PSU-H-LED NIR laser instrument (MDL-N-808-10W; Changchun, China). The temperature produced from nanoparticles was recorded using the PeakTech 3450 thermal imager instrument (Bangkok, Thailand). For this experiment, different concentrations of FCPDA were mixed with DDW and exposed for 10 min to 2 W/cm^2^ of laser light at an 808 nm wavelength. Every 20 s, the temperature was measured and plotted. The photothermal profile of 400 µg/mL at 0.5, 1, 1.5, and 2 W/cm^2^ laser power densities was also captured and shown. Furthermore, five ON/OFF laser cycles with a 10 min exposure time each were used to test the materials’ photothermal stability.

### 2.6. In Vitro Cytotoxicity and Cell Uptake Studies

HepG2 (hepatocellular carcinoma) was purchased from the American Type Culture Collection (Rockville, MD, USA). The HepG2 cells were cultured using Dulbecco’s Modified Eagle Medium (DMEM). All cell media were phenol-free, contained 10% fetal bovine serum, and 100 IU/mL penicillin at 100 mg/mL. Cells were maintained in a humidified incubator at 37 °C under 5% CO_2_ atmosphere.

Using a hemocytometer, cells were trypsinized and manually counted. At a density of 1 × 10^4^, the cells were seeded into 96-well tissue culture plates and given 24 h to adhere using the proper cell culture media (100 µL). For Dox concentration, dependent toxicity was studied by treating various concentrations of Dox for both Dox alone and Dox loaded nanoparticles. The FCPDA and Dox@FCPDA nanoparticles with varying concentrations were added to 96-well plates. For photothermal cancer therapy, the nanoparticles with cells were treated under NIR laser for 10 min at 808 nm with 2 W/cm^2^. Using the Prestoblue^®^ cell viability Assay, the toxicities were assessed after a 24 h incubation period. Each well’s absorbance was assessed using wavelengths of 570 and 600 nm.

HepG2 cells were seeded onto German cover glass slips at a density of 5000 cells/cm^2^ and cultured for 24 h. Cells were then exposed to 20 µg/mL of Dox@FCPDA nanoparticles and incubated for 3 and 6 h. Each well was then filled with 500 µL of 4% formaldehyde solutions, which were then incubated for 10 min. For 10 min, the cells were counterstained with DAPI (nucleus) dye. The glass coverslips were then carefully removed from each well and mounted onto glass slides using a Vector shield after the wells had been cleaned with PBS. With the aid of fluorescence microscopy (Nikon Eclipse Ti, Genova, Italy), the uptake was verified.

## 3. Results and Discussion

### 3.1. Synthesis and Characterization of FCPDA Nanoparticles

**Scheme 1:**
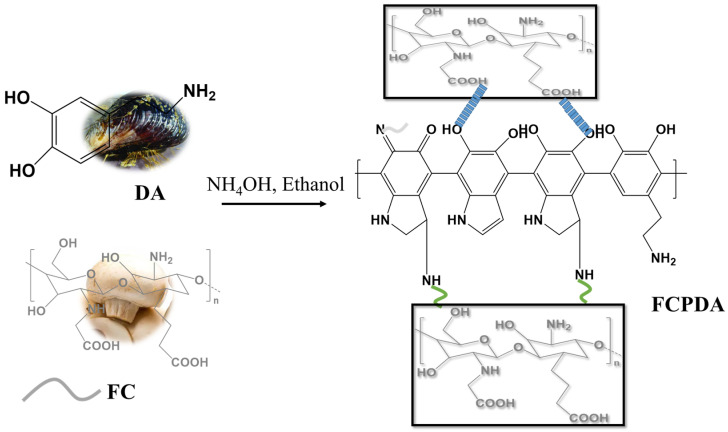
Schematic representation of formation of FCPDA nanoparticles and Dox@FCPDA and its chemistry.

In general, PDA nanoparticles are easily prepared under basic conditions. The use of ammonia solution with ethanol can control the formation of the spherical size of PDA nanoparticles, as described by previously published work [26]. In this work, FC biopolymer can be used for the preparation of functionalized PDA nanoparticles. Owing to the presence of amino functional groups, FC can easily be bonded with DOPA structures during the formation of PDA. Scheme 1 represents the schematic reaction between the FC and PDA of FCPDA nanoparticles. The FC has abundant amino (-NH_2_) and carboxylic (-COOH) functional groups. It is well-known that amine functional groups can easily interact with PDA via coupling reactions. Similarly, the amino functional groups on FC can also be reacted with the DOPA structure of PDA via coupling reactions, thereby stabilizing the FCPDA nanoparticles. Furthermore, the carboxylic groups on FC also involved electrostatic and H-bonding with PDA. Therefore, the multiple bonding interactions between FC and PDA can improve the stability of FCPDA nanoparticles. 

**Figure 1:**
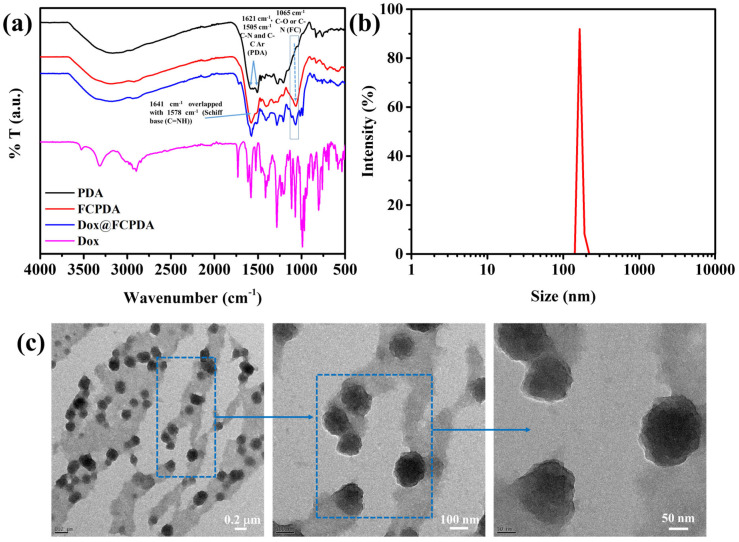
(**a**) FTIR spectra, (**b**) DLS average hydrodynamic size of FCPDA nanoparticles, and (**c**) TEM images of FCPDA nanoparticles.

The resulting FCPDA nanoparticles were characterized by FTIR spectra to know the functional group existence and its bonding. As seen in Figure 1a, bare PDA nanoparticles showed major peaks at 1505 and 1621 cm^−1^ that were attributed to C-N bending and C-C stretching vibrations of indole aromatic rings. FCPDA nanoparticles showed a broad peak between 3000 and 3400 cm^−1^ that is attributed to -OH and -NH stretching of FC and PDA structures. Peaks at 1578 and 1515 cm^−1^ are attributed to H-bonded carboxylate (-COOH) and stretching and bending vibrations of the NH and C=C indole structure of PDA, respectively [27]. Furthermore, there was a broad peak at 1641 cm^−1^ with the combination of 1578 cm^−1^ due to Schiff base bonds between amino groups of FC and the carbonyl group of PDA. Moreover, a small peak at 1261 cm^−1^ corresponded to the stretching vibration of catechol hydroxyl C-O and/or C-N. In addition to In addition, there was a characteristic peak at 1065 cm^−1^ due to the C-O peak of the C-OH groups of FC, suggesting successful conjugation of FC on PDA structure [15]. The typical TEM image of FCPDA nanoparticles is shown in Figure 1c. The spherical morphology of FCPDA nanoparticles was demonstrated. The size of nanoparticles from TEM images is about 112 nm. The size distributions of the FCPDA nanoparticles were further characterized by DLS studies (Figure 1b). It can be observed that the FCPDA nanoparticles are spherical, with an average diameter of 115 nm. The size of FCPDA nanoparticles obtained from the DLS study agrees with TEM results.

### 3.2. UV-Vis-NIR Absorption Spectra

**Figure 2:**
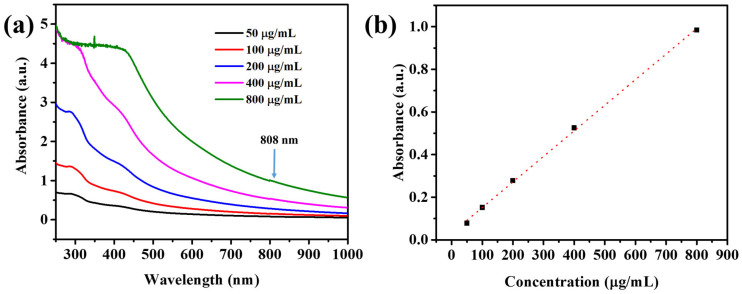
(**a**) UV-Vis-NIR absorption of FCPDA nanoparticles (50–800 µg/mL), and (**b**) graph shows the linear connection between absorbance and FCPDA nanoparticle concentration at a wavelength of 808 nm.

Further, to explore the possible photo-thermal material, it was referred to as an optimal biological window due to its deeper tissue penetration depth and should exhibit a strong capacity for absorption in the NIR light range. As a result, the UV-Vis-NIR absorption curves of FCPDA NPs aqueous solutions at varied concentrations were examined. Figure 2a shows that the absorbance at 808 nm (NIR window) of the FCPDA nanoparticles increased along with their concentration, which suggests that the FC functionalization with PDA did not influence photothermal capacity. Therefore, it is conceivable that when concentration rose, the photo-thermal capacity would rise as well. Figure 2b is a graph that shows the linear correlation between wavelength at 808 nm and FCPDA concentrations, further demonstrating that concentration increased with the increase in absorbance value at a wavelength of 808 nm. 

### 3.3. Photothermal Properties of FCPDA Nanoparticles

**Figure 3:**
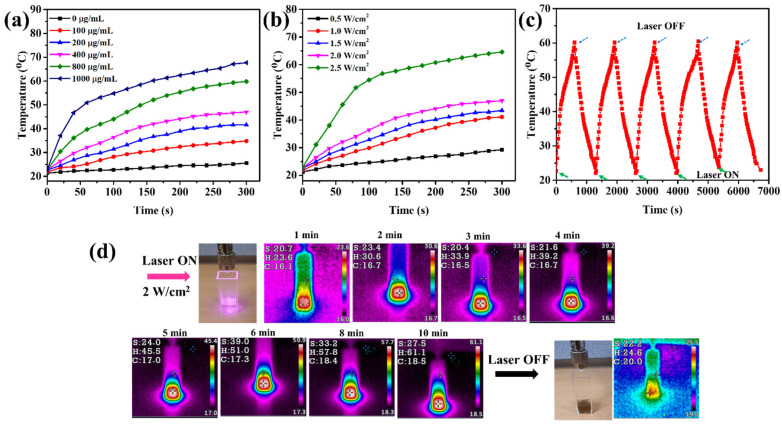
Photothermal heating kinetic graphs of FCPDA nanoparticles at (**a**) different concentrations, (**b**) different power densities, (**c**) heating and cooling cycles with or without NIR laser treatment, and (**d**) infrared photographs of FCPDA nanoparticle illumination under 808 nm laser at 2 W/cm^2^ with respect to time.

By measuring the temperature after being exposed to 808 nm laser irradiations at a power density of 2.0 W/cm^2^, the effectiveness of the various concentrations of FCPDA nanoparticles for in vitro photothermal conversion was assessed. As seen in Figure 3a, after being exposed to 808 nm laser irradiations for 5 min, the temperature of the DDW did not considerably change. The presence of nanoparticles influences the temperature. The temperature rose from 34 to 67 °C and the concentration from 100 to 1000 µg/mL in under 5 min of irradiation at constant power density (2 W/cm^2^). When exposed to laser radiation, the PDA-based biomaterials exhibit noteworthy photothermal conversion properties. The temperature of the FCPDA nanoparticles in an aqueous solution increased to 47 °C when the laser power density was set to 2.0 W/cm^2^, which is sufficient to kill cancer cells.

As can be shown in Figure 3b, the FCPDA nanoparticles were further studied for their photo-thermal conversion across a range of power densities (0.5 to 2.5 W/cm^2^) at a constant concentration of 400 µg/mL. When the FCPDA nanoparticles in an aqueous solution reached a temperature of 60.1 °C, the power density of the laser increased to 2.5 W/cm^2^, which was sufficient to destroy the tumor cells. Five heating/cooling cycles of the FCPDA nanoparticles were carried out to examine the photo-thermal stability, as shown in Figure 3c. Within ten minutes of laser illumination at a power density of 2 W/cm^2^, the maximum temperature for 400 µg/mL FCPDA nanoparticle concentrations was around 60.1 °C. When the laser was turned off, the temperature was significantly reduced to room temperature. More proof of the FCPDA nanoparticles’ strong photothermal properties include the following. After five cycles, there was no discernible temperature drop, further demonstrating the FCPDA nanoparticles’ excellent photothermal stability and their suitability for application as long-term releasing heat in photothermal cancer therapy. In a recent study, Zhang et al. developed multi-responsive PDA-functionalized MXene nanoparticles for drug delivery and antibacterial activity [28]. They found that a temperature of 52.9 °C was produced by applying a power density of 2.0 W/cm^2^ for 10 min. Similarly, Xing et al. reported the development of mesoporous PDA nanoparticles for chemophotothermal therapy. Under laser irradiation of 2 W/cm^2^ at 808 nm for 20 min, a temperature of 62 °C was produced in a dispersion of 1000 μg/mL nanoparticles [29]. Li et al. also developed NIR-active PDA-based nanobombs for on-demand drug release to treat tumor therapy, and observed a temperature of 52.6 °C at a concentration of 0.1 mg/mL nanoparticles and 5 W/cm^2^ for 5 min of incubation [9]. The amount of heat generated by PDA nanoparticles depends on the concentration of nanoparticles, irradiation time, and laser intensity. In our study, we found that a maximum temperature of 60.1 °C was achieved within ten minutes of laser illumination with a power density of 2 W/cm^2^, using a nanoparticle concentration of 400 µg/mL. These results suggest that our findings could be more beneficial for photothermal cancer therapy. 

Further, to support temperature produced from FCPDA nanoparticles, thermal imaging photographs of the FCPDA nanoparticles were taken using a thermal imaging camera when the FCPDA nanoparticles (400 µg/mL) were illuminated by an IR laser using 808 nm laser light at 2.0 W/cm^2^. Figure 3d shows how the temperature of the FCPDA nanoparticles rose from 23.6 °C to 61.1 °C as the length of the laser irradiation increased (up to 10 min). Importantly, after 10 min of radiation, the temperature could increase to up to 61.1 °C, which is strong enough to destroy the tumor cells completely. According to PDA, donor-acceptor molecular pair configurations may reduce the energy bandgap and enhance the electron delocalization of aromatic catechol rings on PDA structure, thereby improving light absorption [30]. As a result, the functionalization of FC biopolymer had no impact on the photothermal property of PDA nanoparticles, confirming past findings and bolstering the idea that PDA can boost the biostability of a drug delivery system. 

### 3.4. Dox Loading and Release

**Figure 4:**
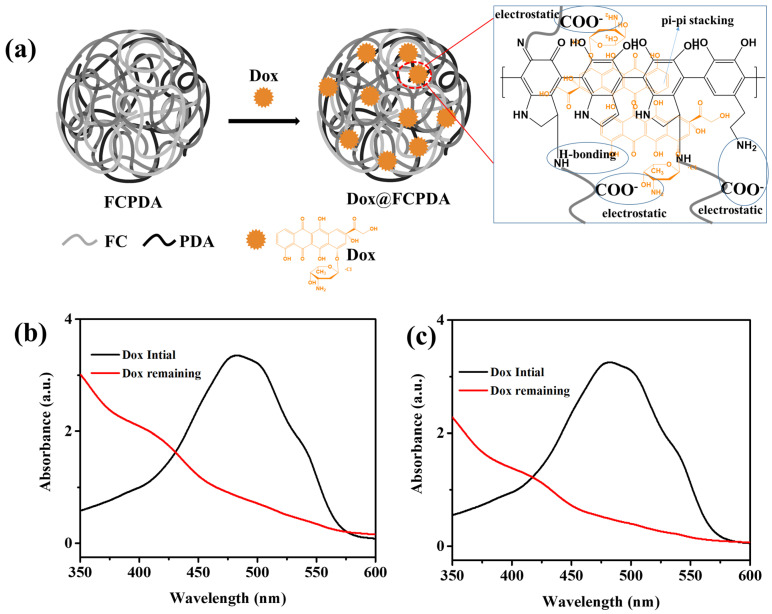
(**a**) Schematic representation of Dox loading to FCPDA nanoparticles and their interactions between Dox and FCPDA nanoparticles, (**b**) UV-Vis absorption spectra of Dox in DDW before and after encapsulation into (**b**) PDA and (**c**) FCPDA nanoparticles.

The drug loading and encapsulation efficiency mainly depended on the functional groups in nanoparticles and drugs. As from Figure 4b, the Dox loading and encapsulation efficiencies were calculated for bare PDA nanoparticles. The %DL and %EE for Dox-loaded bare PDA nanoparticles were about 13% and 68%. Due to the presence of catechol phenolic groups and amino groups in the PDA nanoparticles, the dox could easily be encapsulated into PDA nanoparticles via the formation of pi-pi stacking, H-bonding, and electrostatic interactions between Dox and PDA since Dox have the amino, phenolic, and hydroxyl functional groups. In general, mesoporous silica nanoparticles can enhance the drug loading of Dox because of their larger surface area and abundant functionalities. The PDA-based nanoparticles have shown similar drug loading because they could involve electrostatic, pi-pi stacking, and H-bonding interactions with drugs. Busa et al., developed Dox-loaded PDA-coated Copper-substituted MSNs for dual cancer therapy [31]. The nanoparticles allowed encapsulation of the Dox to be 8%. The surface functionalization can enhance the Dox loading to the nanoparticles. Wei et al. developed Dox loaded mesoporous silica nanoparticles decorated with PDA for cancer therapy [32]. The %DL achieved was as much as 16.25%. The ionic liquid-based PDA nanoparticles showed maximum Dox to be 10.79%. In another report, polyzwitterion and PDA-coated MSNs showed a maximum DL (%) of 8.8% [33]. Most of the reports showed that lower drug loading was achieved for the PDA-based nanoparticles [31,32,33]. Considering the abundant carboxylic functional groups on FC biopolymer, more Dox was encapsulated into FCPDA nanoparticles. In general, carboxylic functionalized nanoparticles could enhance the loading of Dox via the formation of electrolyte complexation between carboxylic groups (nanoparticles) and amino groups of Dox [34]. Similarly, the FC also contains abundant carboxylic groups, which have negative zeta potential at neutral pH conditions. We expect the FC functionalization with PDA nanoparticles to enhance the Dox loading. In this study, the FCPDA nanoparticles show that the maximum %DL and %EE were calculated as 19.3% and 80.2%, respectively [Figure 1c]. As seen in Figure 1a, the Dox was easily encapsulated with FCPDA nanoparticles via the formation of electrostatic (-COO^−^ groups of FCPDA and amino groups of Dox), pi-pi stacking (between phenyl groups from FCPDA and Dox), and H-bonding (amino, hydroxyl, and carboxylic groups from FCPDA and hydroxyl, amino, carbonyl, and carbonyl groups from Dox) interactions. Therefore, the FC functionalization on PDA nanoparticles also significantly improved its %DL and %EE as compared with bare PDA nanoparticles and other reported works [31,32,33]. Further, to confirm the Dox interactions with FCPDA nanoparticles, the Dox@FCPDA nanoparticles were characterized by FTIR spectra. As seen in Figure 1a, the DOX bands were observed at 2929 (C-H), 1720 (C=O), 1621 and 1572 (N-H), 1410 (C-C), and 1064 (C-O) cm^−1^. These peaks were also present in the spectrum of Dox@FCPDA nanoparticles, suggesting the successful encapsulation of Dox into FCPDA nanoparticles. 

**Figure 5:**
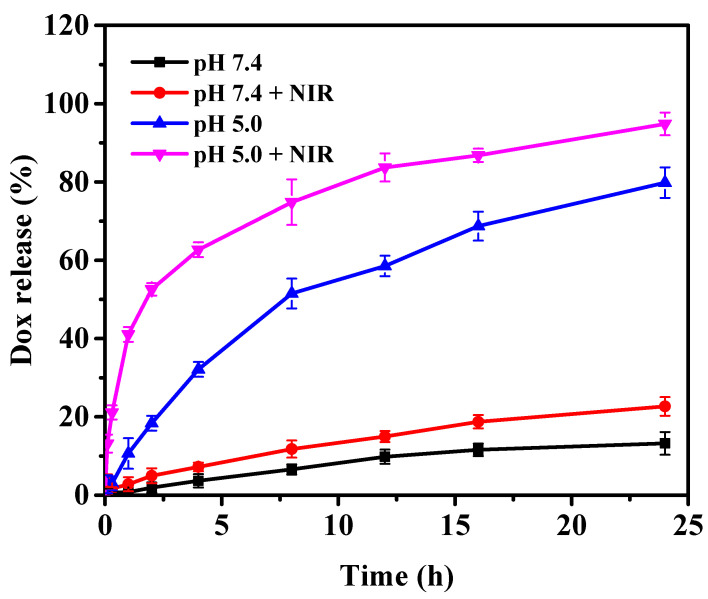
% Dox release at pH 7.4 and pH 5.0 with and without NIR irradiation for 10 min at 808 nm, 2 W/cm^2^ (*n* = 3).

**Figure 6:**
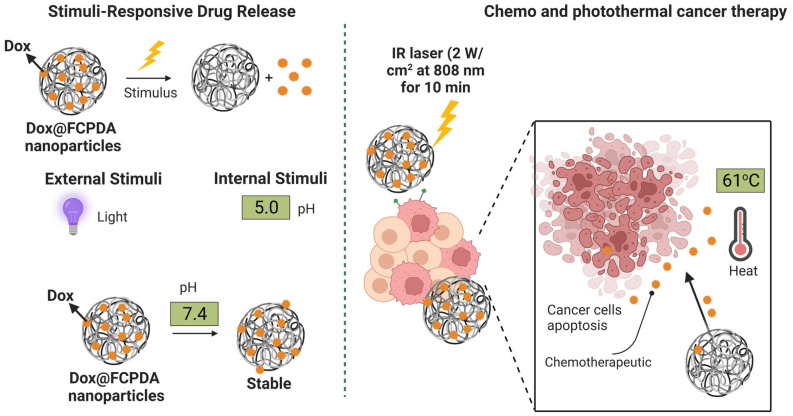
Schematic representation of stimuli-responsive (pH and NIR active) Dox release from FCPDA nanoparticles, and its influence on the cancer cell apoptosis with effect to the synergetic action (Dox and heat) when irradiated at 808 nm for 10 min (2 W/cm^2^) (The image was created in Bio render).

Although there are various potential drug delivery systems for chemotherapy for cancer, PDA nanoparticles are advantageous for cancer therapy due to their PTT properties [35,36]. Considering the photothermal property of PDA and sensitive behavior of FC functionalization, we expect its dual trigger release properties. The PDA-based nanoparticles have shown dual responsive characteristics for the release of the drug. The PDA nanoparticles show not only photothermal properties but also pH-sensitive properties, since the nanoparticles stabilized through pi-pi stacking and abundant amino functional groups, which are easily destabilized under tumor acidic pH conditions, thereby allowing drug release to be triggered. In another study, the functionalization of PEG also showed pH-sensitive drug release for cancer therapy [37,38,39]. In this work, the functionalization of FC with PDA enhanced the Dox release under tumor acidic pH conditions because of the dissociation of electrostatic bonds between Dox and FCPDA. In order to confirm the pH-sensitive property of nanoparticles, the in vitro release of Dox was performed in both tumor and normal physiological pH conditions (pH 7.4 and pH 5.0). A phosphate-buffered saline (PBS) with pH 7.4 was chosen to mimic the typical physiological milieu of the human body. A PBS with a pH of 5.0 was used to represent the tumor microenvironment. For this, in vitro Dox release from FCPDA nanoparticles was evaluated in both pH 7.4 and pH 5.0 with and without NIR treatment at 808 nm with 2 W/cm^2^ (Figure 5). PDA-based nontherapeutic formulations show maximum drug release occurred in the tumor microenvironment (pH 5.0) due to partially destroyed pi-pi interactions in PDA. In this study, a slow release profile was observed. Only 13% of the Dox was released in 24 h in a physiological milieu of the human body (pH 7.4), whereas 22% Dox was released in the presence of NIR laser illumination (808 nm), which suggests the FCPDA nanocarriers had no side effects on normal tissue (Figure 6). A fast Dox release occurred in the tumor microenvironment (pH 5.0), i.e., 79% within 24 h. This can be explained based on the dissociation of FCPDA nanostructures in an acidic environment due to the protonation of amino groups and the dissociation of pi-pi stacking bonds. When NIR illumination was at 808 nm, 94% of Dox was released due to its pH and photothermal effects (Figure 6). 

### 3.5. In Vitro Cytotoxicity and Cellular Uptake Study

**Figure 7:**
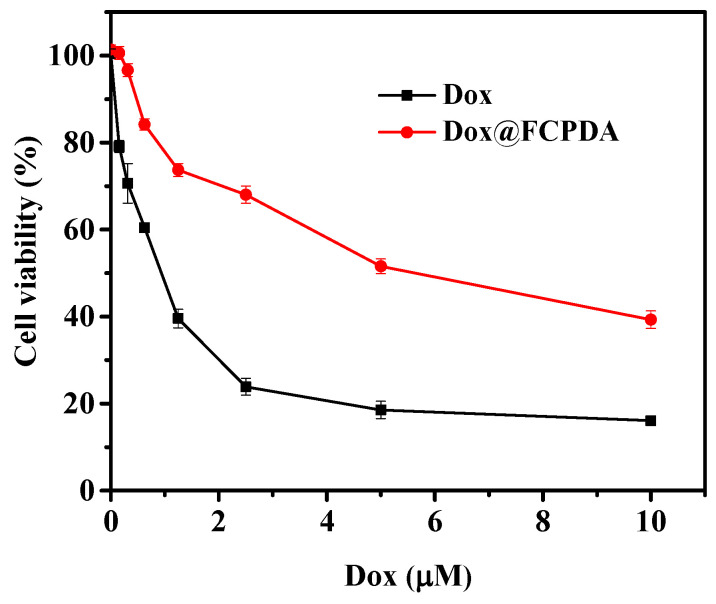
In vitro cytotoxicity of HepG2 cancer cells treated with various concentrations of Dox alone and Dox-loaded FCPDA nanoparticles.

Initially, the PrestoBlue assay was utilized to evaluate the in vitro cytotoxicity of Dox alone and Dox-loaded FCPDA nanoparticles without NIR laser. The cytotoxic effects of both free Dox and Dox@FCPDA on HepG2 cells were assessed, revealing concentration-dependent cytotoxicity for Dox and Dox@FCPDA nanoparticles (Figure 7). The half maximal inhibitory concentration (IC50) values were determined for both Dox and Dox@FCPDA, with IC50 values of 0.84 ± 0.016 and 7.321 ± 1.284 μM, respectively.

**Figure 8:**
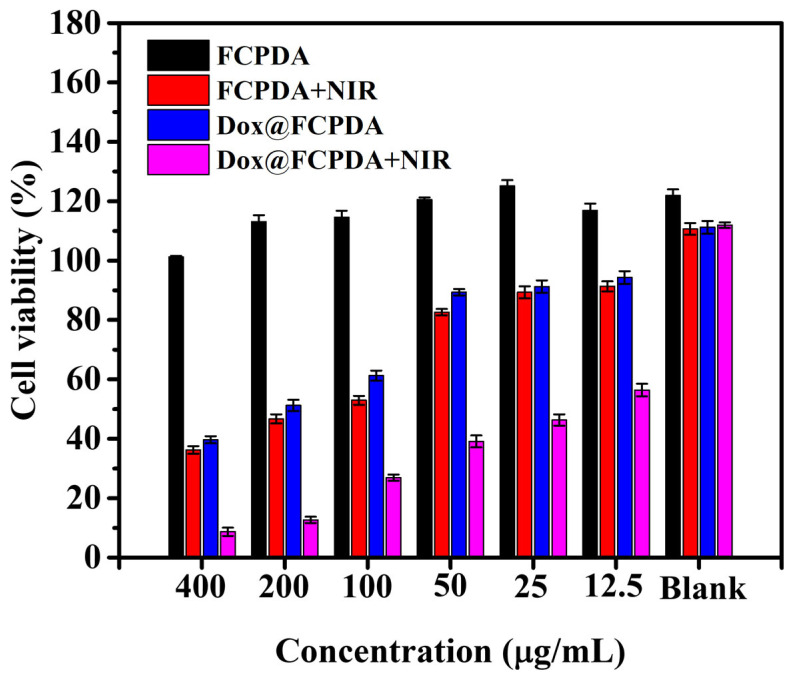
In vitro cytotoxicity of HepG2 cancer cells treated with various concentrations (400–12.5 µg/mL) of FCPDA and Dox@FCPDA nanoparticles with and without NIR laser treatment (808 nm, 2 W/cm^2^, and 10 min).

Further, the cytotoxicity of FCPDA and Dox@FCPDA nanoparticles (400–0 µg/mL) with and without NIR laser treatment was examined against HepG2 cancer cells using Prestoblue assay. The outcomes demonstrated a concentration-dependent trend in the relative cell viability of HepG2 cells after 24 h incubation with FCPDA and Dox@FCPDA nanoparticles. As seen in Figure 8, the HepG2 cells treated with FCPDA nanoparticles (400–0 µg/mL) without NIR laser treatment showed 100% cell viability. In contrast, the HepG2 cells treated with FCPDA nanoparticles with NIR laser treatment at 808 nm for 10 min showed cytotoxicity (~36% viability). The Dox@FCPDA nanoparticles treated with HepG2 cells showed cytotoxicity with 39% cell viability. In contrast, cells treated with Dox@FCPDA nanoparticles irradiated with NIR laser at 808 nm for 10 min indicated more significant cytotoxicity (~8% cell viability) due to synergetic therapeutic benefits such as Dox drug and heat, which can quickly kill the HepG2 cancer cells (Figure 6). This result implies that the Dox@FCPDA nanoparticles could induce the photothermal effect with different NIR laser sources.

**Figure 9:**
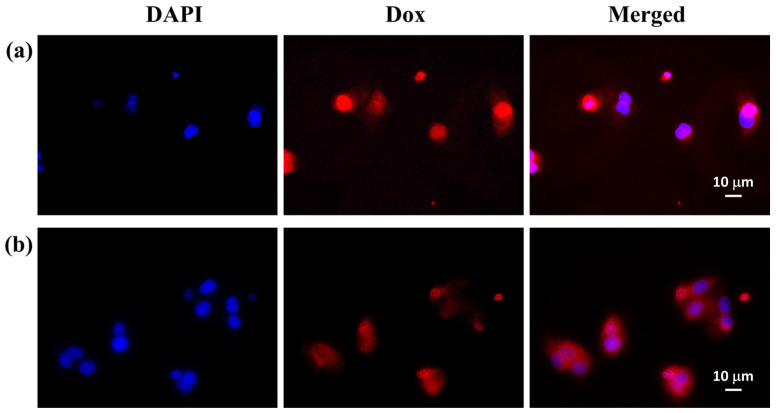
Florescence microscopy images of HepG2 cancer cells treated with Dox@FCPDA nanoparticles (**a**) 3 h and (**b**) 6 h incubation (blue color indicates the cells stained with DAPI and red color indicates Dox).

Fluorescence microscopy analysis was used to track the Dox@FCPDA nanoparticles’ intracellular uptake activity. HepG2 cells were treated with Dox@FCPDA nanoparticles (20 µg/mL) and then incubated for 3 and 6 h, respectively, to assess endocytosis and subcellular localization. Fluorescence microscopy was used to detect the presence of internalized nanoparticles in the treated cells after incubation. As seen in Figure 9, the Dox molecules’ distinct red fluorescence signal was visible inside and around the nucleus, indicating that the Dox@FCPDA nanoparticles were successfully absorbed by the HepG2 cancer cells. The red fluorescence signal intensities can be seen in the merged images to be larger in the cells incubated for 6 h (Figure 9b) compared with the cells incubated for 3 h (Figure 9a), suggesting that the Dox release was significantly increased after 6 h incubation. The Dox@FCPDA nanoparticles, however, demonstrated improved cell uptake ability and could be quickly absorbed by HepG2 cells in an incubation period. They could also release the loaded Dox from the FCPDA nanoparticles under intracellular cancer cells due to their acidic pH levels. 

## 4. Conclusions

In conclusion, we have developed Dox@FCPDA nanoparticles for combined chemo and photothermal therapy applications. Our results demonstrate that FCPDA nanoparticles exhibit maximum Dox loading with %DL and %EE values of 19.3% and 80.2%, respectively. These nanoparticles also exhibit pH-responsive drug release properties. Moreover, our FCPDA nanoparticles exhibit high photothermal conversion efficiency under NIR laser irradiation at 808 nm with a concentration of 400 μg/mL and power density of 2 W/cm^2^. In vitro cell studies further revealed that Dox@FCPDA nanoparticles enhance cytotoxicity towards HepG2 cancer cells due to their synergistic performance in chemo-photothermal therapy. Additionally, Dox@FCPDA nanoparticles were readily internalized by HepG2 cells, demonstrating their potential for use in synergistic chemo-photothermal combination therapies in the field of novel cancer treatments. Despite this, our proof-of-concept study has revealed that our newly developed carrier has the potential to function as a photothermal chemotherapy vehicle for Dox to treat cancer. Additional in vivo studies are required to significantly improve the delivery effectiveness of synergistic performance of Dox@FCPDA nanoparticles for cancer therapy.

## Data Availability

Not applicable.
